# Suppression of Esophageal Cancer Stem-like Cells by SNX-2112 Is Enhanced by STAT3 Silencing

**DOI:** 10.3389/fphar.2020.532395

**Published:** 2020-12-16

**Authors:** Dan-dan Xu, Su-hong Chen, Peng-jun Zhou, Ying Wang, Zhen-dong Zhao, Xia Wang, Hui-qing Huang, Xue Xue, Qiu-ying Liu, Yi-fei Wang, Rong Zhang

**Affiliations:** ^1^ Guangdong Food and Drug Vocational College, Guangzhou, China; ^2^ College of Life Science and Technology, Jinan University, Guangzhou, China; ^3^ State Key Laboratory of Oncology in South China and Collaborative Innovation Center for Cancer Medicine, SunYat-Sen University Cancer Center, Guangzhou, China; ^4^ College of Food Science and Technology, Zhongkai University of Agriculture and Engineering, Guangzhou, China

**Keywords:** SNX-2112, Hsp90, STAT3, suppression, esophageal cancer stem‐like cells

## Abstract

Many studies have demonstrated that cancer stem cells (CSCs) or tumor-initiating cells (TICs) are responsible for tumor cell proliferation, chemotherapy resistance, metastasis, and relapse in various cancers. We, and others, have previously shown that the signal transducer and activator of transcription 3 (STAT3) signaling pathway is responsible for CSCs and TICs growth. Recent reports have indicated that the heat shock protein 90 (Hsp90) is also essential for the survival of CSCs and TICs. SNX-2112 is an Hsp90 inhibitor. However, it remains unclear whether proliferation of esophageal cancer stem-like cells (ECSLCs) is suppressed by SNX-2112 with knockdown of STAT3 (shSTAT3). Here, we explored the association between SNX-2112 with shSTAT3 and the suppression of ECSLCs growth. We found that the expression level of both STAT3 and p-STAT3 was higher in clinical esophageal cancer tissue than in the adjacent normal tissue, using western blot and qPCR analysis. Furthermore, differential expression analysis demonstrated that STAT3 was overexpressed in clinical specimens. We demonstrated that SNX-2112 inhibited cancer cell proliferation, decreased *ABCB1* and *ABCG2* gene expression levels and reduced the colony formation capacity of ECSLCs, which was enhanced by STAT3 silencing. Flow cytometry analysis revealed that the combination of SNX-2112 and shSTAT3 significantly induced apoptosis and cell cycle arrest at G2/M phase in ECSLCs. Levels of proliferation pathway proteins, including p38, c-Jun N-terminal kinase (JNK), and extracellular signal–regulated kinase (ERK) which were also client proteins of Hsp90, were also reduced. In addition, SNX-2112 with shSTAT3 inhibited the proliferation of ECSLCs *in vivo*. Finally, STAT3 overexpression eliminated the apoptotic and antiproliferative effects of SNX-2112 on ECSLCs. Hence, these results provide a rationale for the therapeutic potential of the combination of SNX-2112 with shSTAT3 in esophageal cancer, and may indicate new targets for clinical intervention in human cancer.

## Introduction

Esophageal cancer is the seventh most common cause of cancer-associated death globally (Siegel et al., 2020). The mortality rate for patients with esophageal cancer is high, and the 5-years survival rate is less than 20%, even in developed countries like the United States (Siegel et al., 2020). According to epidemiology and pathology, esophageal cancer is of two types: esophageal squamous cell carcinoma (ESCC) and esophageal adenocarcinoma (EAC) (Middleton et al., 2018). ESCC is the most common type of esophageal cancer, and is highly prevalent in East Asia, East Africa, and South America (Middleton et al., 2018). Although clinicians and researchers have made progress with treatments for esophageal cancer, most patients are diagnosed at a later stage, when metastasis has occurred (Itskoviz et al., 2019). A lack of effective chemotherapeutic drugs is responsible for the high mortality rate.

According to cancer stem cells (CSCs) hypotheses, cancer cells from small subpopulations are responsible for tumor cell proliferation, invasion, metastasis, relapse, and resistance (Jeter et al., 2011; Noh et al., 2012; Song et al., 2017). In addition, CSCs have the ability to self-renew (Noh et al., 2012). To-date, CSCs have been isolated and identified in many types of solid tumors, including breast, prostate, brain, colorectal, and pancreatic cancers (Sancho et al., 2015). CSCs are identiﬁed primarily through their aldehyde dehydrogenase (ALDH) activity, along with several surface markers, including CD44, CD90, and CD133 (Zhao et al., 2011; Hang et al., 2012; Wang et al., 2017). In esophageal cancer cells, CD44 ^+^ cells are considered to be CSCs (Zhao et al., 2011). In previous studies, esophageal cancer stem-like cells (ECSLCs) were isolated and examined, demonstrating high expression levels of stem-like markers, as well as tumor sphere formation and tumorigenesis induction (Xu et al., 2016).

Molecular chaperones, such as heat shock protein 90 (Hsp90), interact with their client proteins to stabilize them and assist in their folding. They play an essential role in cell stress process, such as during starvation and at low temperatures, to prevent their client proteins from mis-folding (Hoter et al., 2018). Hsp90 helps its client proteins to recover from cell stress, either by protein refolding or by degradation to restore homeostasis. Hsp90 is implicated in carcinogenesis by promoting cancer cell proliferation, as well as inhibiting cell death pathways (Hoter et al., 2018). The client proteins of Hsp90 are associated with the hallmarks of cancer, and inhibition of Hsp90 has been considered an efficient strategy for cancer therapy (Wang et al., 2010). Emerging evidence demonstrates that Hsp90 inhibition is effective in targeting CSCs (White et al., 2016; Nolan et al., 2017). Extracellular Hsp90 upregulates stemness markers, promotes self-renewal, and enhances tumor sphere growth in prostate cancer patients, which suggest that extracellular Hsp90 is a modulator of CSCs in prostate cancer (Nolan et al., 2017). Inhibition of extracellular Hsp90 using the monoclonal antibody mAb4C5 reduced the activity of breast CSC *in vitro* and significantly inhibited the growth of breast cancer cells *in vivo* (Stivarou et al., 2016). Additionally, inhibition of Hsp90 using novel C-terminal inhibitors KU711 and KU757 completely prevented the self-renewal of head and neck squamous cell carcinoma CSCs (Subramanian et al., 2017). At the same time, they found that suppression of Hsp90 effectively targeted the functionality of thyroid CSCs, which prevented their migration and invasion (White et al., 2016). In our previous studies, the Hsp90 inhibitor SNX-2112 demonstrated antitumor activity by induction of apoptosis and cell cycle arrest of melanoma cells, and inhibiting tumor growth *in vivo* (Liu et al., 2012a; Liu et al., 2012b; Wang et al., 2014). In addition, it exerted its inhibitory effect on various cancer cells by binding to the N-terminal adenosine triphosphate binding site of Hsp90 (Wang et al., 2015). Moreover, SNX-2112 is a more effective agent compared to the classic Hsp90 inhibitor, 17-allylamino-17-demethoxygeldanamycin (17-AAG). For example, SNX-2112 inhibited melanoma cell proliferation in a dose-dependent manner to a more significant level than 17-AAG and the IC_50_ values of 17-AAG and SNX-2112 at 48 h were 1.25 and 0.16 μM, respectively (Liu et al., 2012b). The inhibitors of Hsp90 are listed in Table 1. These results encouraged us to investigate the effects of SNX-2112 on ECSLCs.

**TABLE 1:** Summary of Hsp90 inhibitors reaching clinical trials.

Hsp inhibitor	Class of compound	Cancer type	Phase	Ref
17-AAG	Geldanamycin analogue	Prostate, papillary, and clear cell RCC,melanoma V600EHarboring BRAF mutation	Phase II	(Solit et al., 2008) NCT00118092
17-DMAG	Geldanamycin analogue	Solid tumors, AML	Phase I	(Lancet et al., 2010) NCT00088868
IPI-504	Geldanamycin analogue	GIST progression after TKI NSCLC progression on EGFR inhibitor	Phase III Phase I/II	(Sequist et al., 2010; Wagner et al., 2013) NCT00606814
AUY922	Resorcinol derivate	NSCLC progression on chemotherapy	Phase II	(Garon et al., 2013) NCT01854034
AT-13387	Resorcinol derivate	CRPC progression on abiraterone	Phase II	(Shapiro et al., 2015) NCT01685268
STA-9090	Resorcinol derivate	Solid and hematological malignancies	Phase I	(Acquaviva et al., 2014) NCT00858572
SNX-5422	Purine and purine-like analogue	HER2^+^ tumor types NSLC, esophagogastric, breast	Phase I/II	(Rajan et al., 2011) NCT01848756
NVP-HSP990	Purine and purine-like analogue	Advanced solid tumors	Phase I	(Spreafico et al., 2015) NCT00879905
XL -888	Purine and purine-like analogue	Advanced solid tumors	Phase I	(Jhaveri et al., 2012) NCT00796484

RCC, renal cell carcinoma; CRPC, castrate-resistant prostate cancer; GIST, gastrointestinal stromal tumors; NSCLC, non-small cell lung cancer; TKI, tyrosine kinase inhibitor; EGFR, epidermal growth factor receptor.

Accumulating evidence has demonstrated that STAT3 signaling is associated with the proliferation of CSCs or TICs (Chung et al., 2013; Kulesza et al., 2019). STAT3 is a key transcription factor with many functions in normal stem cells, CSCs, and embryonic stem cells (ESCs) (Zhong et al., 1994; Marotta et al., 2011; da Hora et al., 2019; Zhang et al., 2019). This transcription factor is necessary for maintaining mouse ESCs in an undifferentiated state, and is regulated via a Myc-dependent mechanism (Wong et al., 2018). STAT3 has been identified as an oncogene, and activated STAT3 can mediate cellular transformation (Bromberg et al., 1999). Indeed, its regulation is complex as it is involved in many signaling pathways, in many types of cancer cells (He et al., 2018; Lin et al., 2018). STAT3 is constitutively activated by tyrosine phosphorylation in numerous cancers, including esophageal cancer (Chen et al., 2013). Aberrant expression of STAT3 has been implicated in malignant transformation and tumor progression (Xiong et al., 2012). Further studies have demonstrated that STAT3 is overexpressed in CSCs in brain cancer, leukemia, and breast cancer (Hosea et al., 2018; Shastri et al., 2018; Han et al., 2019). Consistently, STAT3 level was higher in melanoma samples and it supported maintenance of melanoma CSCs. It is suggested that STAT3 could serve as a potential target to impair tumor progression or recurrence (Kulesza et al., 2019). STAT3 is also overexpressed in ECSLCs (Xu et al., 2016).

Notably, Hsp90 is important for the functional competence of STAT3 which governs the tumor microenvironment and cancer progression (Bocchini et al., 2014; Cho et al., 2019). The association between Hsp90 and STAT3 was identified in tumor cells, and is necessary for STAT3 phosphorylation, dimerization, and nuclear translocation, all of which contribute to cancer cell survival (Chatterjee et al., 2007; Bocchini et al., 2014). Hsp90 inhibition may simultaneously suppress both Hsp90 functionality and STAT3 signaling activity (Cho et al., 2019). However, it remains unclear whether STAT3 inhibition influences the anti-tumor activity of Hsp90 inhibitors.

In the present study, we investigated the molecular mechanisms underlying the ECSLCs-targeting effects of STAT3 knockdown combined with SNX-2112. We found that ECSLCs proliferation was inhibited by combination treatment. We also demonstrated that the expression level of p-STAT3 is higher in clinical esophageal samples than in paired normal cells. Moreover, the levels of Hsp90 client proteins were significantly reduced in ECSLCs after STAT3 depletion and treatment with SNX-2112. Finally, knockdown of STAT3, along with SNX-2112 administration, inhibited the growth of ECSLCs *in vivo*.

## Materials and Methods

### ECSLCs Culture

SNX-2112 was synthesized in our laboratory according to a known procedure, with purity of the compound >98.0% (Barta et al., 2008). SNX-2112 was dissolved in DMSO and 10 mM SNX-2112 stock solution was stored in 4°C. Eca109 cancer cells were obtained from the Cell Bank of the Chinese Academy of Science. Immortalized human esophageal epithelial cells (HEEC) were purchased from BNBIO (Beijing, China). ECSLCs were isolated and identified according to our previous study (Xu et al., 2016). After they were isolated, the ECSLCs were maintained in serum-free DMEM/F12 medium supplemented with B27 supplement (1:50) (Invitrogen, Carlsbad, CA, United States), 20 ng/ml epidermal growth factor (EGF) (Pepro Tech, Inc. Rocky Hill, United States), 20 ng/ml basic fibroblast growth factor (bFGF) (Pepro Tech, Inc. Rocky Hill, United States) using ultra-low attachment plates (Coring, NY, United States). Clinical esophageal samples were provided by the Cancer Center of Sun Yet-Sen University in October 2014. Primary ESCC tumors and adjacent normal tissues were obtained from eight patients who underwent surgical treatment. The specimen was same with our previous studies (Xu et al., 2016). The patients provided written informed consent to participate in the study. The use of the clinical specimens for research purposes was approved by the Ethics Committee of Guangdong Food and Drug Vocational College.

### Cell Viability Assay and Ki-67 Labeling

The proliferation of ECSLCs was evaluated using the MTT (3-(4, 5-dimethylthiazol-2-yl)-2, 5-diphenyltetrazolium bromide) assay. The ECSLCs were stably transduced with lentiviral constructs carrying shGFPctrl or shSTAT3 for 72 h. Next, approximately 4 × 10^3^ cells were seeded into 96-well culture plates and cultured overnight in DMEM/F12 medium containing 20 ng/ml EGF, 20 ng/ml bFGF, and B27 (1: 50). The following day, to the combination treatment group the cells had been transfected with lentiviral and after 72 h, the cells were exposed to SNX-2112 for 24 h. To the shGFPctrl and shSTAT3 groups ECSLCs were treated with lentiviral constructs for 72 h and cell viability was measured. To SNX-2112 group the ECSLCs were treated with SNX-2112 and cell viability was measured. The MTT assay solution (10 µL) was added to each well, and the plates were incubated at 37°C for 4 h. The MTT final concentration in treatment media was 5 mg/ml. Then the medium was removed, and the formazan crystals were dissolved in DMSO. The absorbance (A) was measured at 570 nm using a microplate reader (Elx800, Biotek). To detect the proliferation of ECSLCs using the Ki-67 marker, the cells were treated with SNX-2112 and shSTAT3 for 24 h. The next day, the cells were incubated with the Ki-67 antibody (1:1,000, Cell Signaling Technology, #9449) at room temperature for 1.5 h after the cells were blocked with 10% goat serum at room temperature for 1.0 h. The cells were then washed three times with Tris-buffered containing 01% Tween-20 for 5 min. Next, the cells were incubated with FITC-conjugated secondary antibody (goat anti-mouse/rabbit) (1:8,000, Beyotime, Haimen, Jiangsu, China) at 37°C for 0.5 h. The cells were then washed three times with Tris-buffered saline (TBS) for 5 min and photographed using a fluorescence microscope (Nikon, Japan).

### Construction of shRNA-Expressing Vectors and Transfection

The STAT3-specific shRNA carrying lentiviral vectors with GFP were constructed (Genechem, Shanghai, China) in order to silence the expression of STAT3. The shRNA sequence were as follows, shSTAT3, forward primer, 5′-GCC​ATT​GGC​CGG​AAT​TAG​CGA​ACG​GT-3′, reverse primer, 5′-CCGGTTAAAG GTT​CGA​CTT​CCA​AGG​TA-3′; shSTAT3-1, forward primer, 5′-GCT​AAA​CCG​GTG​CCA​GCT​GAG​TTC​CCA-3′, reverse primer, 5′-TTG​GCG​TAA​GGT​TCG​TAC​AGT​TGG​TCC-3′; shGFP-ctrl, forward primer, 5′-GTC​GGA​AGT​CCC​AAG​GTT​AGT​CCG​T-3′, reverse primer, 5′-GGT​TAC​GTA​AGG​TCC​GAC​TGG​AC-3′. The complementary oligonucleotides encoding shRNA were designed and packaged. The sequences of plasmids were verified by sequencing. The cells were transfected with a serum-free medium in the presence of 8 μg/ml polybrene to improve the knockdown efficiency for 4 h. After 4 h, the serum-free medium was replaced by normal medium. The silence efficiency of shSTAT3 was evaluated in our previous studies (Xu et al., 2016).

### Ribonucleic Acid Sequencing and Quantitative PCR Analysis

The total RNA was extracted from the tumor tissue using Trizol reagent (Invitrogen, CA, United States) following the manufacturer’s procedure. RNA integrity was assessed using the RNA Nano 6000 Assay Kit of the Bioanalyzer 2,100 system (Agilent Technologies, CA, United States). After total RNA was extracted, the ribosomal RNA in the total RNA was completely removed using the Ribo-Zero Magnetic Gold Kit (illumina, United States). Subsequently, the RNA was broken into fragments and connected to the sequencing connector using NEB Next Ultra Directional RNA Library Prep Kit for Illumina (NEB, United States) following the manufacturer’s procedure. The cDNA libraries were sequenced by Novogene Technologies (Beijing, China) using an Illumina HiSeq Xten platform. Raw data generated by sequencing were recorded. The specific processing steps were as follows: removal of short sequences; removal of contaminated information which cannot be determined; removal of low-quality reads. At the same time, Q20, Q30, and GC content of the clean data were calculated. All of the analyses were based on the clean data with high quality. For Quantitative PCR (qPCR) analysis, total RNA was isolated using TRIzol reagent (Invitrogen) and reverse transcribed into cDNA using a standard protocol (TaKaRa Biotechnology, Dalian, China). qPCR was carried out using the Bio-Rad system (Hercules, CA, United States) and the TaqMan system (Applied Biosystems, Foster City, CA, United States). The PCR primers were synthesized (Sangon Biotech, Shanghai, China). The primer sequences for PCR were as follows: ABCB1, forward primer 5′-CAG​CTG​TTG​TCT​TTG​GTG​CC-3′, reverse primer 5′-TGG​CAA​TGC​GTT​GTT​TCT​GG-3′. ABCG2, forward primer 5′-TGGTGT TCCTTGTGACACTG-3′, reverse primer 5′-TGA​GCC​TTT​GGT​TAA​GAC​CG-3′. GAPDH, forward primer 5′-ATT​CCA​CCC​ATG​GCA​AAT​TC-3′, reverse primer 5′-TGG​GAT​TTC​CAT​TGA​TGA​CAA​G-3′. Each sample was analyzed in triplicate. Gene expression level data analysis was performed according to the 2^−ΔΔCt^ method using GAPDH expression as the control. The following PCR procedure was used on the Light Cycler: 95°C for 5 s, 60°C for 5 s, followed by 42 cycles of 95°C for 15 s and 60°C for 1 min, in a 10 μl reaction volume.

### Western Blot Analysis

Cells were collected and lyzed with 1% SDS containing phenylmethylsulfonyl fluoride (PMSF), and protein concentration in the samples was measured using the BCA protein assay kit (Beyotime, Haimen, China). Samples containing 20 µg protein were separated by SDS-PAGE and transferred onto 0.22 µm polyvinylidene fluoride membranes (Millipore, Billerica, MA, United States). Membranes were blocked with 5% skimmed milk in TBS containing 0.05% Tween-20 (TBST) for 1.5 h and were incubated with primary antibodies [Bcl2, Cell Signaling Technology (CST), c15071; Bax, CST, #5023; p38, abcam, ab32142; p-p38, abcam, ab126425; ERK, CST, #4695; *p*-ERK, CST, #4370; JNK, CST, #9252; *p*-JNK, CST, #9255; STAT3, CST, #9139; p-STAT3 (Tyr705), CST, #9145; GAPDH, CST, #5174] at 4°C overnight. The following day, the membranes were washed three times with 0.1% TBST. The membranes were incubated with HRP-conjugated secondary antibody (1: 8,000, goat anti-mouse antibody or goat anti-rabbit antibody) for 1.5 h. The signals on the membranes were developed with ECL reagent (Thermo Scientific, Waltham, MA, United States).

### Flow Cytometry Assay

For the apoptosis assay, cells were treated with shSTAT3 and SNX-2112. They were then harvested and washed with PBS for 5 min. The cells were incubated with 5 µl of binding reagent and 5 µl of Annexin V-APC (KeyGen BioTech, China). After 30 min, cells were washed three times with PBS and stained with 5 µl of 7-AAD (KeyGen BioTech, China) for 25 min at room temperature, according to the manufacturer’s instructions. The experiments were repeated three times. All data were analyzed and calculated using FlowJo software.

### TUNEL Assay

ECSLCs apoptosis was analyzed post-treatment using the terminal deoxynucleotidyl transferase–mediated deoxyuridine triphosphate nick end labeling (TUNEL) method, using the *in-situ* cell death detection kit (Beyotime, Haimen, China), according to the manufacturer’s instructions. Paraffin sections were dewaxed in xylene for 10 min, followed by soaking in a series of descending alcohol concentrations (EtOH for 5 min, 90% EtOH for 2 min, 70% EtOH for 2 min, distilled water for 2 min). The sections were incubated with 20 mg/ml proteinase K for 20 min at 30°C in an incubator. The sections were washed with PBS three times for 5 min and soaked in 3% H_2_O_2_ to block endogenous peroxidase. The sections were incubated with TUNEL reagents. The percentage of TUNEL-labeled cells was determined at a magnification of ×400 by counting 500 cells in a random field.

### Colony Formation Assay

For colony formation, according to our previous study (Xu et al., 2016), a 6-well plate was coated with a bottom agar layer containing DMEM/F12 medium supplemented with recombinant human EGF, recombinant human bFGF, and B27 supplement (1:50). The top agar layer contained a single cell suspension of 1 × 10^3^ ECSLCs transfected with shSTAT3 in DMEM/F12 medium supplemented with EGF (20 ng/ml), bFGF (20 ng/ml), B27 supplement (1:50), and 0.2 μM SNX-2112. After 14 days, the cells were stained with 0.5% crystal violet for 30 min at 37°C, and colonies of more than 50 cells were counted as positive.

### Animal Experiments and Immunohistochemical Analysis

The study protocol was approved and conducted according to the Jinan University Laboratory Animal Ethics Committee. Five-week-old male Balb/c nude mice were purchased from the Animal Center of Huafukang (Beijing, China). These mice were divided randomly into four groups containing six animals each. ECSLCs containing shSTAT3 were washed with PBS three times and counted. The cells (1 × 10^5^) were resuspended in 150 μl PBS, mixed with an equal volume of Matrigel (BD Biosciences, MA, United States), and injected subcutaneously into the neck area of each nude mouse. The tumor size was measured every three days, and the tumor volume was calculated as *L* × *W* ^2^ × 0.5 (mm^3^; *L* indicates length; *W* indicates width). After seven days, SNX-2112 was intraperitoneally injected into mice. In addition, in control group the mice were inoculated 1 × 10^5^ cells and seven days later equal volume of PBS was intraperitoneally injected, which was same as the experiment group treatment. Every other day the mice were administrated 10 mg/kg of SNX-2112. After drug treatment for 2 weeks, animals from each group were euthanized, and the tumors were harvested and measured to determine their weight.

For IHC analysis, tumor tissue and adjacent non-tumor esophageal tissue were fixed in 4% paraformaldehyde and paraffin-embedded. Then, the tissues were sectioned at 3–4 µm thickness and deparaffinized. The sections were dewaxed and subjected to antigen retrieval (0.01 M Citrate buffer pH 6.0 and 0.01 M Tris-HCI buffer pH 9.0, respectively). Endogenous peroxidase was blocked using 0.6% H_2_O_2_ for 30 min, followed by washing steps with TBS. Sections were treated with 10% normal goat serum in 0.1% TBST for 10 min for non-specific antibody binding. Tissue sections were incubated with primary antibodies [p-ERK, CST, #4695, 1:100; *p*-AKT (Ser473), CST, #4060, 1:100; STAT3, CST, #9139; p-STAT3 (Tyr705), CST, #9145]. Secondary antibody incubation was performed at room temperature for 90 min. Chromogen reaction was performed by incubating with diaminobenzidine (DAB) for 5 min. Slides were counterstained with hematoxylin, dehydrated, and permanently mounted using standard procedures. IHC quantification was performed on three randomly selected high power fields (HPF) per slice. Quantification of apoptotic cells was performed manually. Integrated optical density (iod) of Ki-67^+^, *p*-AKT and *p*-ERK were quantified using Image pro-Plus software by applying the appropriate pixel threshold equally on all selected pictures and using measure function to calculate the covered area. Data is represented as % of covered area.

### Statistical Analysis

The SPSS19.0 program (SPSS Inc., Chicago, IL, United States) was used for statistical analysis. The data are presented as the means ± SD of at least three independent experiments. Student’s *t*-test was used for two-group comparisons. *p* < 0.05 was considered to indicate a statistically signiﬁcant difference.

## Results

### STAT3 Level Was Higher in Clinical Esophageal Cancer Samples

STAT3 is constitutively activated in numerous types of human cancers, including ECSS, and plays a key role in regulating proliferation, chemo-resistance, and relapse.

**FIGURE 1:**
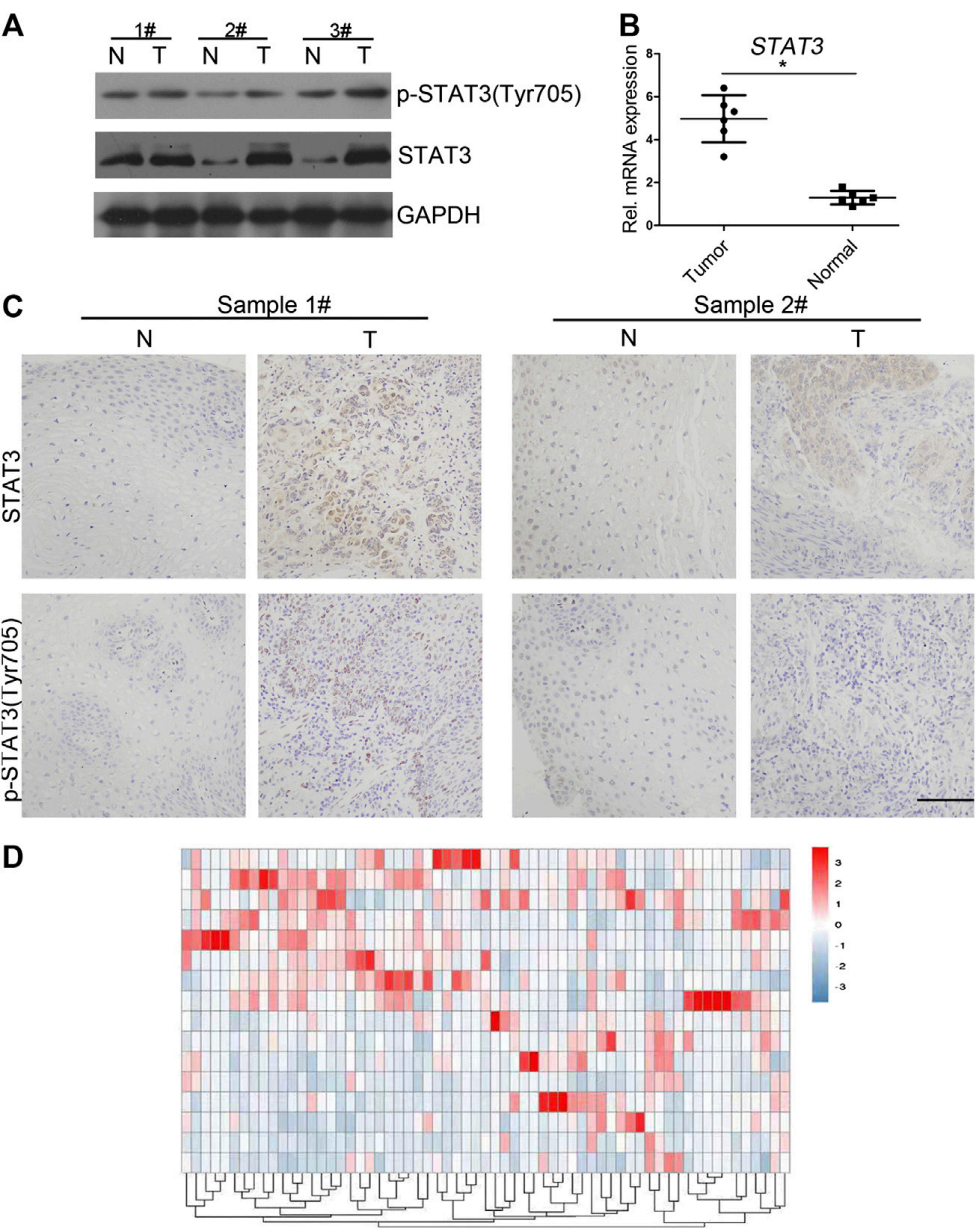
Expression of STAT3 and p-STAT3 in clinical esophageal cancer samples **(A)** Western blot analysis of the STAT3 expression in clinical samples. STAT3 expression in tumor tissues was compared with that in the adjacent normal tissues. T, tumor tissues; N, normal. **(B)** qPCR analysis of the STAT3 expression level in clinical samples. mRNA expression levels of STAT3 in clinical tumor tissue were compared with that in normal tissue. The experiments were repeated three times independently (mean ± SD). **(C)** Expression levels of STAT3 and p-STAT3 in paraffin sections of clinical tumor tissue were analyzed by immunocytochemistry. Representative images of various clinical esophageal cancer specimen sections from eight independent cases and the percentage of samples showing high STAT3 and p-STAT3 expression levels are provided. Scale bar, 50 μm. **(D)** Heat maps of differential expressions of mRNA in clinical esophageal cancer samples.

However, its clinical significance and biological role in the regulation of proliferation remains unexplored. Western blot analysis indicated that both STAT3 and p-STAT3 (Tyr705) protein levels were increased in human ECSS samples compared with adjacent non-tumor tissue samples (Figure 1A). qPCR analysis showed that remarkably higher level of STAT3 was detected in clinical cancer samples (Figure 1B). IHC results were also consistent with western blot results regarding levels of STAT3 and p-STAT3 (Tyr705) (Figure 1C). Additionally, we compared gene expression profiles of eight esophageal cancer cases with those of normal adjacent tissues using RNA sequencing. Differential expression analysis demonstrated that STAT3 was overexpressed in six out of eight cases (Figure 1D). Together, these results demonstrated that STAT3 level is higher in esophageal cancer cells, and STAT3 may play an important role in regulating the proliferation of esophageal cancer cells.

### shSTAT3 Enhanced SNX-2112 Efficacy by Inhibiting ECSLCs Colony Formation

**FIGURE 2:**
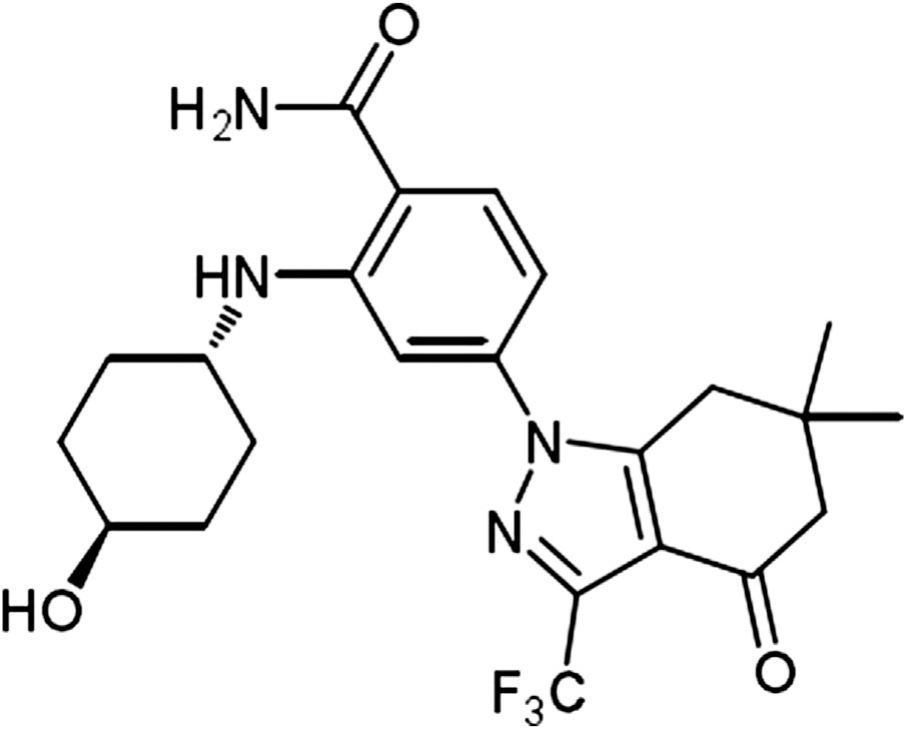
The chemical structure of SNX-2112.

**FIGURE 3:**
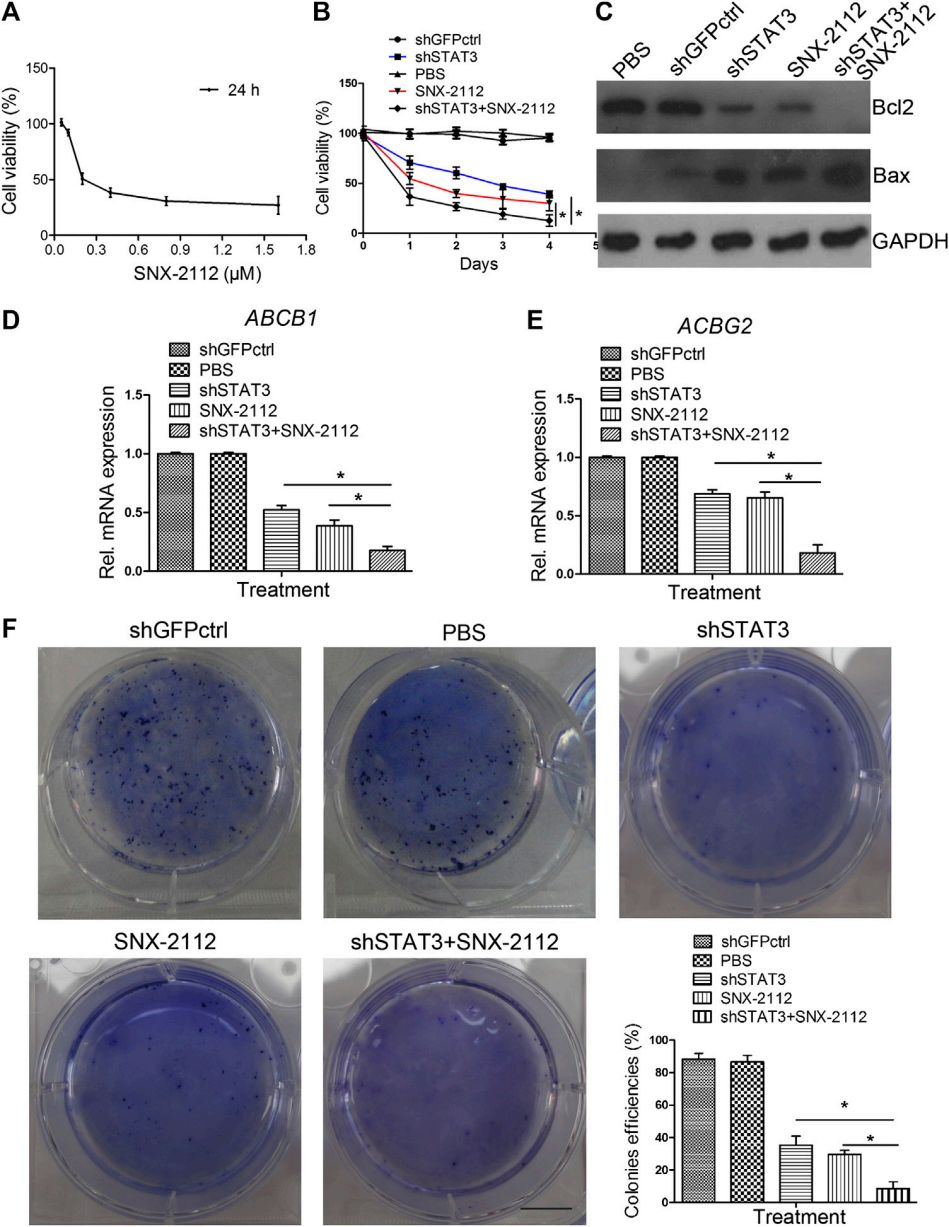
The inhibition of colony formation capacity of ECSLCs by combination treatment of SNX-2112 with shSTAT3 **(A)** Cell viability of ECSLCs treated with SNX-2112 for 24 h. ECSLCs were treated with various concentrations of SNX-2112 fro 24 h and cell viability was assessed using the MTT assay. **(B)** Proliferation curve analysis of ECSLCs after treatment with SNX-2112 and shSTAT3. ECSLCs were stably transduced with lentiviral constructs carrying shGFPctrl and shSTAT3. After 72 h, ECSLCs were treated with 0.2 μM SNX-2112 for 24 h. Then, ECSLCs proliferation was evaluated by MTT assay. The experiments were repeated three times independently. **(C)** Western blot analysis of Bcl2 and Bax expression levels in ECSLCs post treatment with shSTAT3 for 72 h and 0.2 μM SNX-2112 for 24 h. GAPDH was used as a loading control. **(D,E)** qPCR analysis of *ABCB1* and *ABCG2* in ECSLCs. After transfection with shSTAT3 for 72 h, ECSLCs were treated with 0.2 μM SNX-2112 for 24 h. Vertical bars represent mean ± SD. **(F)** The colony forming capacity of ECSLCs was tested using soft agar plates. Approximately 1 × 103 ECSLCs post treatment with shSTAT3 and 0.2 μM SNX-2112 were allowed to grow for approximately 14 days and formed colonies were stained by 0.5% crystal violet and counted. The experiments were repeated three times independently. Scale bar, 10 mm.

The chemical structure of SNX-2112 is shown in Figure 2. First the cell toxicity of SNX-2112 was studied using HEEC. According to our previous studies (Liu et al., 2012a; Liu et al., 2012b), HEEC were cultured with 20, 40, and 80 μM SNX-2112 for 24, 48, and 72 h. As in the Supplementary Figure S1, no significant cell toxicity was observed. In addition, both western blot and qPCR assays demonstrated that the silence efficiency of shSTAT3 was higher than shSTAT3-1 (Supplementary Figure S2). Therefore, the shSTAT3 was selected to conduct the following experiments. To investigate the anticancer effects of the combination treatment of SNX-2112 with shSTAT3, ECSLCs viability was analyzed. The ECSLCs was cultured in the presence of a range of concentration of SNX-2112 (0.05, 0.1, 0.2, 0.4, 0.8,1.6 μM) for 24 h. The viability of ECSLCs was inhibited in a concentration-dependent manner (Figure 3A), and the IC50 value of SNX-2112 at 24 h was 0.19 μM. So for the convenience in the following studies the treatment time and concentration of SNX-2112 was 24 h and 0.20 μM, respectively. SNX-2112, along with shSTAT3, remarkably suppressed cell proliferation compared with shSTAT3 and SNX-2112 alone (Figure 3B). Furthermore, a significant downregulation of the mitochondrial protein Bcl2 level was observed with the combination therapy. Bax expression level in the combination treatment group was higher than that in the shSTAT3 and 0.20 μM SNX-2112 alone treatment groups (Figure 3C). In addition, the mRNA level of ABC transporter super-family ABCB1 and ABCG2 was significantly reduced following the combination treatment (Figures 3D,E).

We further investigated the colony formation capacity of ECSLCs cultured in DMEM/F12 medium supplemented with recombinant human 20 ng/ml EGF, recombinant human 20 ng/ml bFGF and B27 (1: 50). The results demonstrated that the combination treatment of SNX-2112 with shSTAT3 significantly decreased the colony formation ability of ECSLCs (Figure 3F). These results indicated that the combination treatment notably reduced the proliferation of the ECSLCs.

### SNX-2112 With shSTAT3 Induced Apoptosis and Cell Cycle Arrest in ESCLCs

**FIGURE 4:**
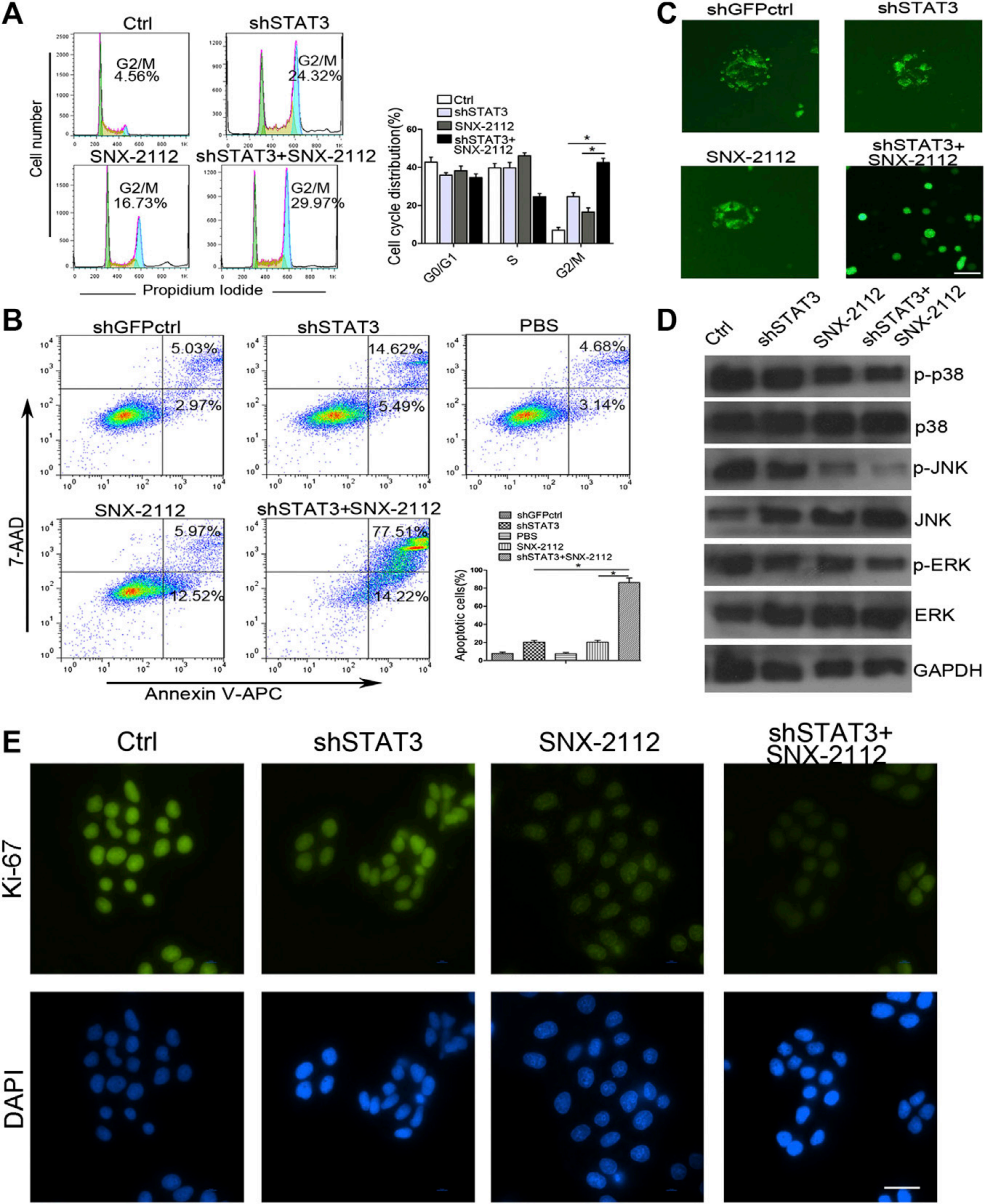
Combination treatment with SNX-2112 and shSTAT3 induced ECSLCs apoptosis and cell cycle arrest **(A)** Flow cytometry analysis of cell cycle of the ECSLCs after treatment with shSTAT3 for 72 h and with 0.2 μM SNX-2112 for 24 h. The cell cycle distribution was analyzed by flow cytometry. Vertical bars represent mean ± SD from three independent experiments. **(B)** The apoptosis of ECSLCs treated with shSTAT3 for 72 h and 0.2 μM SNX-2112 for 24 h was analyzed by flow cytometry. The data were from three independent experiments. **(C)** The sphere formation size detection of ECSLCs. The ECSLCs were treated with shSTAT3 for 72 h and 0.2 μM SNX-2112 for 24 h. Then the ECSLCs were subjected to normal medium for approximately 14 days. The medium was changed every 3 days. Scale bar, 50 μm. **(D)** Western blot analysis of ECSLCs treated with shSTAT3 for 72 h and 0.2 μM SNX-2112 for 24 h. The level of p38, ERK, and JNK were analyzed by western blot. GAPDH was used as a loading control. **(E)** Ki-67 proliferation analysis of ECSLCs treated with shSTAT3 and 0.2 μM SNX-2112 for 24 h. The cells were collected and incubated with Ki-67 antibody. Scale bar, 50 μm.

To explore whether the combination of SNX-2112 with shSTAT3 induced ECSLCs cycle arrest, a flow cytometry assay was conducted. As shown in Figure 4A, the cell cycle was indeed found to be arrested. The relative percentages of G2/M phase cells in the combination treatment, SNX-2112 and shSTAT3 groups were 29.97%, 24.32%, and 16.73%, respectively. In addition, the combination treatment significantly increased the number apoptotic cells compared with the individual treatment (Figure 4B). These results suggest that SNX-2112 and shSTAT3 combination remarkably increased G2/M phase arrest compared with SNX-2112 or shSTAT3 alone.

To further investigate whether SNX-2112 combination with shSTAT3 reduced the proliferation and colony formation size of cancer cells, we performed a gain-of-function analysis *in vitro*, using a shSTAT3 lentiviral vector containing GFP. As shown in Figure 4C, SNX-2112 combination with shSTAT3 significantly decreased the colony formation size of cells.

To explore the molecular changes in cell proliferation, the levels of p38, JNK and ERK which were client proteins of Hsp90, were measured. We found that p-p38 levels were significantly reduced in the combination group compared with SNX-2112 and shSTAT3 alone groups (Figure 4D). Changes were also observed in *p*-JNK and *p*-ERK levels (Figure 4D). In addition, the Ki-67 assay showed that intensity of signal of Ki-67 was reduced, which suggested that the proliferation of ECSLCs was inhibited by the combination treatment of SNX-2112 with shSTAT3 (Figure 4E). Together, these results demonstrate that STAT3 is required for the proliferation of ECSCLs.

### Combination Treatment of SNX-2112 With shSTAT3 Suppressed ECSLCs Tumor Growth *In Vivo*

**FIGURE 5:**
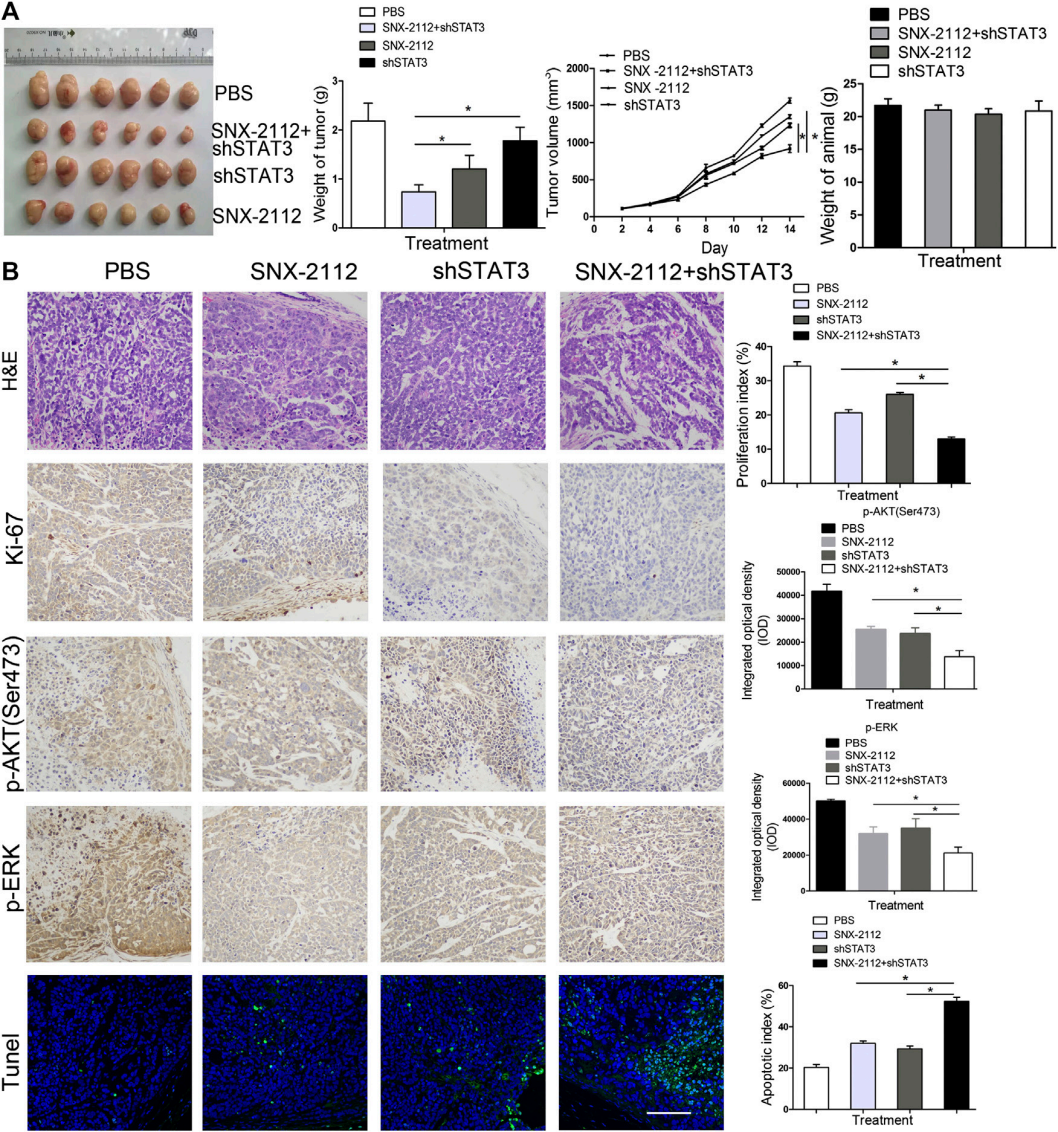
The inhibition of tumorigenicity of ECSLCs by SNX-2112 with shSTAT3 *in vivo* **(A)** Analysis of tumor weight and animal weight. Balb/c nude mice were injected subcutaneously with 1 × 105 ECSLCs transduced with shSTAT3. After 7 days, 10 mg/kg SNX-2112 was intraperitoneally injected into mice. Every other day, the mice were administrated with SNX-2112. Following drug treatment for 2 weeks, animals from each group were euthanized, and the tumors were harvested and measured. Vertical bars represent mean ± SD. **(B)** Expression of Ki-67, *p*-AKT (Ser473) and *p*-ERK proteins in parafﬁn sections of ECSLCs xenografts analyzed by immunocytochemistry. Sections were counter-stained with hematoxylin (blue nuclei). Representative images of various tumor tissue sections were present. TUNEL analysis of cell apoptosis was present. Scale bar, 100 μm.

To investigate the effect of a combination of SNX-2112 with shSTAT3 on esophageal tumor growth *in vivo*, xenograft tumor models with ECSLCs were established. The tumor volume was monitored. The mean weight and volume of tumors in the combination treatment group were smaller than that in the shSTAT3 or SNX-2112 alone groups (Figure 5A). The combination treatment of SNX-2112 with shSTAT3 inhibited the tumor growth (Figure 5A). In addition, the body weight of animal was not significanty reduced in the combination treatment group (Figure 5A). To further investigate the effect of SNX-2112 combination with shSTAT3 on tumor growth, the levels of Ki-67, *p*-ERK and *p*-AKT (Ser473) in the xenograft tumors were examined. It was determined that Ki-67 and *p*-ERK levels were decreased significantly in the combination treatment group. The same effect was observed for *p*-AKT (Ser473) (Figure 5B). TUNEL assay revealed higher levels of cancer cell apoptosis in the combination treatment group (Figure 5B).

### Constitutively Active STAT3 Reduced the Efficacy of SNX-2112 on ECSLCs

**FIGURE 6:**
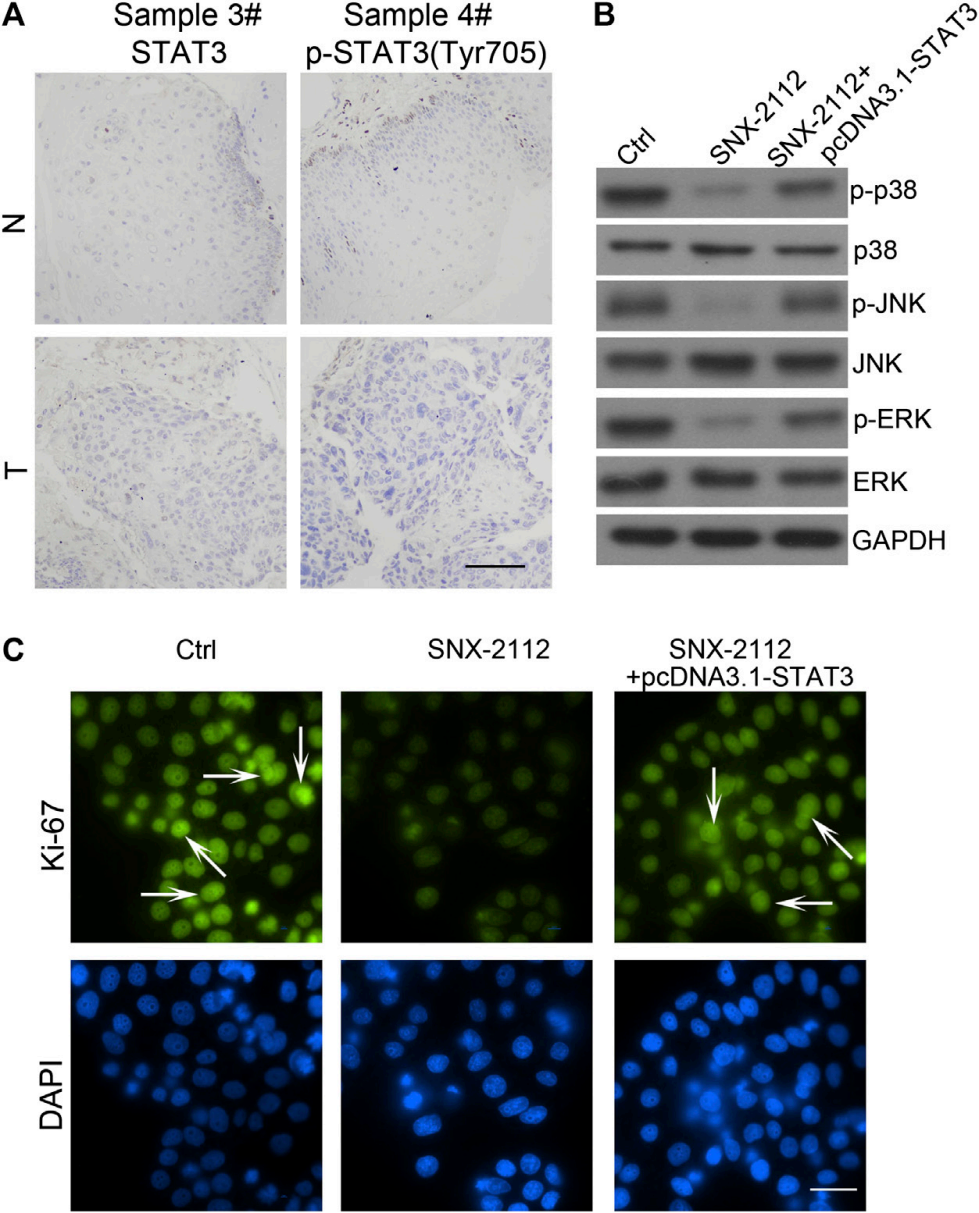
STAT3 overexpression-reducing the efficacy of SNX-2112 **(A)** STAT3 expression was analyzed in tumor specimens compared with adjacent normal tissue. Representative images of various clinical esophageal cancer specimen sections were present. T, tumor tissues; N, normal. Scale bar, 50 μm. **(B)** Western blot analysis of p38, JNK and ERK expression. ECSLCs were transfected with pcDNA3.1-STAT3 vectors for 72 h and then 0.2 μM SNX-2112 for 24 h. GAPDH was used as a loading control. **(C)** The proliferation analysis of ECSLCs treated with pcDNA3.1-STAT3 vectors for 72 h and then 0.2 μM SNX-2112 for 24 h. The cells were collected and incubated with Ki-67 antibody. Scale bar, 50 μm.

Previous studies have demonstrated that STAT3 is overexpressed in many types of tumor cells (Misra et al., 2018; Shastri et al., 2018; Kulesza et al., 2019). To determine whether the STAT3 pathway is required for SNX-2112-induced ECSLCs apoptosis, clinical esophageal cancer samples were collected and the STAT3 and p-STAT3 expression levels were evaluated using IHC. STAT3 and p-STAT3 (Tyr705) levels were higher in tumors than in the adjacent normal tissues (Figure 6A). Western blot assay demonstrated that the expression level of p-p38 was reduced with treatment of SNX-2112, and this effect was reversed by STAT3 overexpression (Figure 6B). Consistently, these results were observed in *p*-JNK and *p*-ERK (Figure 6B). In addition, fluorescence microscopy showed that the proliferation of ECSLCs was inhibited by SNX-2112. However, the proliferation inhibition of the ECSLCs by SNX-2112 was reversed by STAT3 overexpression (Figure 6C). These results demonstrated that STAT3 overexpression reduced the efficacy of SNX-2112 on ECSLCs.

### STAT3 Overexpression Abolished the Apoptotic Effect of SNX-2112 on ECSLCs

**FIGURE 7:**
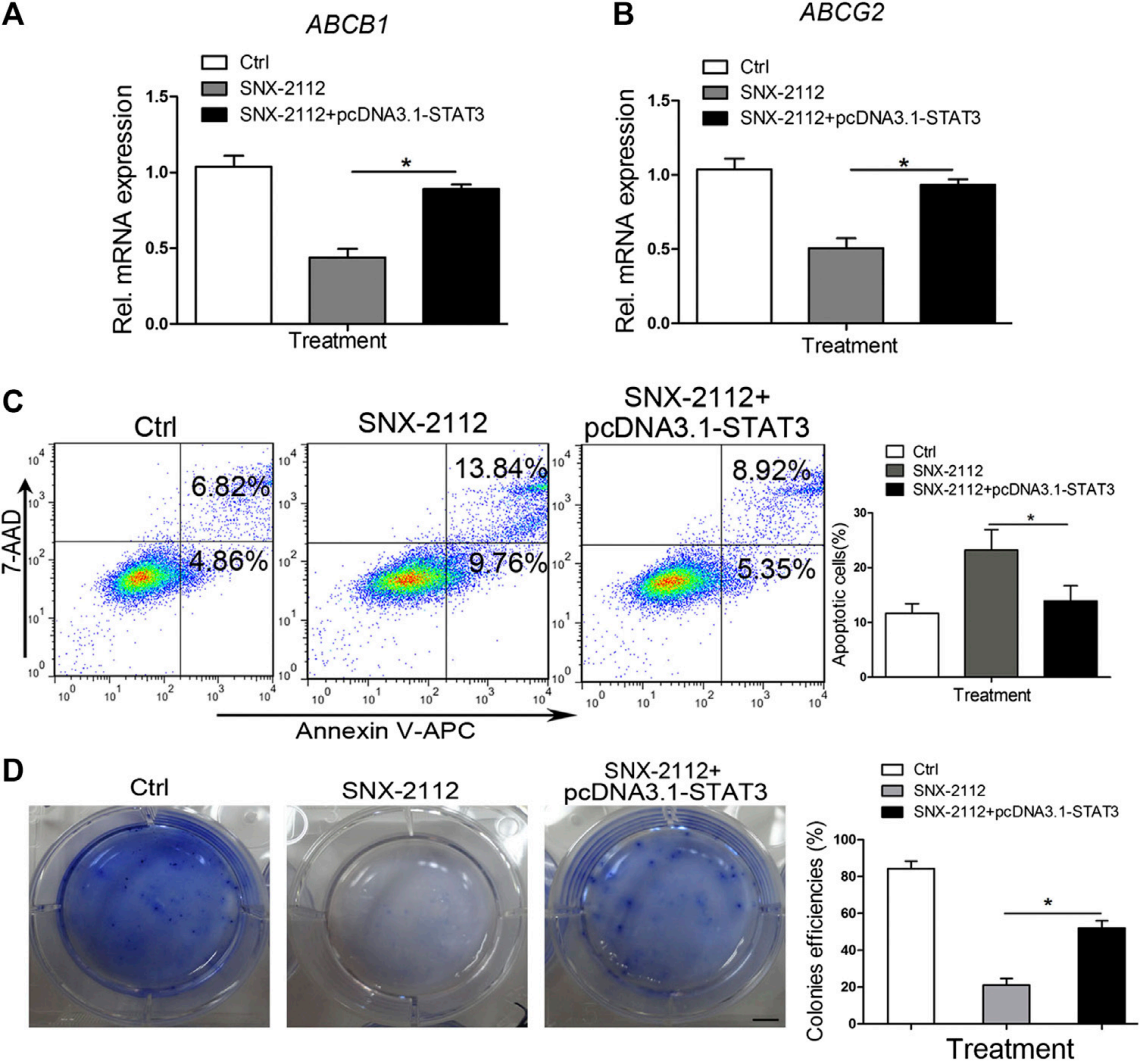
STAT3 overexpression-reversing SNX-2112-induced ECSLCs apoptosis **(A,B)** qPCR analysis of ABCB1 and ABCG2 expression level in ECSLCs. ECSLCs were transfected with pcDNA3.1-STAT3 vectors and after 72 h the ECSLCs were treated with 0.2 μM SNX-2112 for 24 h. **(C)** Flow cytometry analysis of ECSLCs. ECSLCs were treated with pcDNA3.1-STAT3 vectors for 72 h and 0.2 μM SNX-2112 for 24 h. **(D)** The colony formation efficiency analysis of ECSLCs treated with pcDNA3.1-STAT3 vectors and SNX-2112. Approximately 1 × 103 ECSLCs were treated with pcDNA3.1-STAT3 vectors for 72 h and then 0.2 μM SNX-2112 for 24 h. These cells were then cultured on soft agar plates for 14 days, followed by staining with 0.5% crystal violet. Scale bar, 10 mm.

To investigate the effect of STAT3 overexpression on the expression of ABCB1 and ABCG2, the level of ABCB1 and ABCG2 was messured. SNX-2112 reduced the expression level of ABCB1 and ABCG2, which was reversed by STAT3 overexpression (Figures 7A,B). In addition, the percentage of total apoptosis in the control, SNX-2112 and STAT3 overexpression groups was 11.68%, 23.60%, and 14.27%, respectively (Figure 7C). STAT3 overexpression decreased the percentage of the apoptotic cells. To further confirm the biological role of STAT3 in the regulation of ECSLCs proliferation, the colony formation assay was conducted. The colony formation assay demonstrated that STAT3 overexpression increased colony formation ability of ECSLCs (Figure 7D). Collectively, shSTAT3 potentiated the apoptotic effect of SNX-2112 on ECSLCs. Meanwhile, overexpression of STAT3 abolished the apoptosis of ECSLCs induced by SNX-2112. Taken together, these results suggest that SNX-2112 with shSTAT3 inhibited the proliferation of ECSLCs.

## Discussion

CSCs have been isolated and identified in many types of tumor cells, including breast, pancreas, brain, and leukemia (Choi et al., 2014; Ross et al., 2015). CSCs have been found to have stem-like cell properties, such as self-renewal, multilineage differentiation potential, and stemness marker expression (Batlle and Clevers, 2017). More importantly, cancer cells have the abilities of invasion, resistance, metastasis, and relapse, mainly due to the presence of these CSCs (Chen et al., 2011; Sainz and Heeschen, 2013; Williams et al., 2015; Zhao et al., 2015). In this study, we demonstrated that the Hsp90 inhibitor SNX-2112 combined with shSTAT3 suppressed the proliferation of esophageal cancer stem-like cells *in vitro* and *in vivo*. Importantly, the combined treatment of SNX-2112 with shSTAT3 exerted cytotoxic effects against ECSLCs. These results suggest that combined treatment of SNX-2112 with shSTAT3 could be useful for esophageal cancer therapy.

Overexpression of Hsp was found to promote cell survival and protect cellular proteins from the risk of damage or aggregation, such as heat shock, absence of nutrients, and oxidative stress (Pirkkala et al., 2001; Hoter et al., 2018). Hsp90 is known to promote cancer cell survival and anticancer drug resistance by helping their client protein to maintain correct conformation and by enhancing the stability of numerous oncogenic proteins (Taipale et al., 2010; Jego et al., 2013; Le et al., 2018). Hsp90 overexpression is observed in many types of cancer cells, and has emerged as a promising target for anti-tumor drug development (Modi et al., 2011; Le et al., 2018). In addition, several clinical trials are being conducted to evaluate the effectiveness of Hsp90 inhibitors (Modi et al., 2011).

In addition, Hsp90 plays a key role in drug resistance. Cisplatin resistance in bladder cancer-initiating cells was overcome by Hsp90 inhibitor 17-DMAG (Tatokoro et al., 2012). In their study, they found that 17-DMAG simultaneously inactivated both AKT and ERK signaling, which were its client proteins, and synergistically potentiated the cytotoxicity of cisplatin (Tatokoro et al., 2012). The glioma tumor-initiating cells were inhibited in a dose-dependent manner by NVP-HSP990, another Hsp90 inhibitor. NVP-HSP990 disrupted cell-cycle control mechanisms by decreased *CDK2* and *CDK4* levels and increased glioma tumor-initiating cell apoptosis levels (Fu et al., 2013). Furthermore, in aggressive hematological tumors, Hsp90 is still a target for anti-tumor therapy. For example, 17-AAG, an Hsp90 inhibitor, induced apoptosis and disrupted transcriptional functionality of HIF1α, which is a client protein of Hsp90, and 17-AAG decreased the colony formation ability of mouse lymphoma CSCs and human myeloid leukemia CSCs (Newman et al., 2012).

Hsp90 inhibitors were initially designed and developed with the rationale that Hsp90 is overexpressed in cancer cells and emerges as a molecular chaperone to many client proteins (Subramanian et al., 2017). Hsp90-related gene expression is increased in CSCs, which led us to hypothesize that Hsp90 inhibitors may be useful in cancer therapies. Furthermore, Hsp90 inhibitors are more active in tumor cells than in normal tissue, which suggests that these novel inhibitors are likely to preferentially target cancer cell populations and CSCs subpopulations (White et al., 2016; Subramanian et al., 2017). In our study, we demonstrated that treatment with SNX-2112 in combination with shSTAT3 decreased ECSLCs viability and induced ECSLCs apoptosis and cell cycle arrest in G2/M phase. ECSLCs proliferation was significantly inhibited as indicated by reduced colony formation potential and reduced cell viability.

The expression of ABCB1 and ABCG2 was also reduced by treatment with SNX-2112 in combination with shSTAT3. The increased levels of ATP-binding cassette (ABC) transporters is a general mechanism by which cancer cells acquire multidrug resistance in CSCs. High ABCB1 protein expression has been identified as an independent predictor of early recurrence and death for EAC and ESCC patients treated with chemoradiotherapy based on 5-fluorouracil and cisplatin (Zhu et al., 2015). Available studies suggest that expression of several ABC proteins (ABCB1, ABCC2, and ABCG2) correlates with prognosis or response to therapy in esophageal cancer patients (Vrana et al., 2018). Other studies confirmed ABCG2 protein overexpression in the majority (75%) of esophageal cancer tissues and high ABCG2 protein correlates with poor prognosis of ESCC patients (Bharthuar et al., 2014). ABCB1 overexpression has been observed in half of EAC patients whose biopsies were taken before and after treatment with combination of epirubicin, cisplatin and 5-fluorouracil (Narumiya et al., 2011). ABCG2 overexpression accompanied by increased drug efflux rate resulted in resistance of ECA-109 cell line to doxorubicin *in vitro* (Liu et al., 2014). This resistance could be reversed, through downregulation of ABCG2, by administration of epigallocatechin-3-gallate *in vitro* (Liu et al., 2017). Previous studies have already suggested important role for ABCB1 overexpression in paclitaxel and cisplatin-resistance in radio-resistant EC-9706 ESCC model *in vitro*. Interestingly, the resistance to taxane could be blocked by ABCB1 inhibitor verapamil (Wang et al., 2012). In our studies, we found that ABCB1 and ABCG2 levels were reduced by SNX-2112 with shSTAT3.

Accumulating evidence has demonstrated that STAT3 plays an important role in regulating proliferation, survival, relapse, invasion, and self-renewal of CSCs (Kulesza et al., 2019; Zhang et al., 2019). The JAK/STAT3 axis plays an essential role in promoting tumor initiation and radio-resistance in glioma CSCs (Lin et al., 2018). STAT3 suppression reduced stemness-associated gene expression levels and inhibited colony formation capacity of glioma cells, and the intracranial glioma xenograft growth was effectively impaired (Han et al., 2019). Furthermore, in malignant hematopoietic cell disorders, STAT3 was considered a treatment target, and high STAT3 expression was observed in myelodysplastic syndromes. CD34^+^ cell and STAT3 inhibition by antisense oligonucleotides led to reduced viability and increased apoptosis in leukemic cell lines (Shastri et al., 2018). The targeted delivery of the STAT3 modulator reduced expression levels of several stemness genes, including *MYC*, *BCL2*, *EGFR*, and *MMP9,* and caused a reduction of CD44+/CD24-breast CSCs (Misra et al., 2018). In our previous study, we found that miR-181b and STAT3 interacted and their interaction was key for the proliferation of ECSLCs (Xu et al., 2016). In this study, we found that p-STAT3 (Tyr705) was upregulated in clinical esophageal cancer samples. In addition, we found that the Hsp90 inhibitor combination with shSTAT3 significantly suppressed the proliferation of ECSLCs and induced apoptosis. SNX-2112 with shSTAT3 inhibited ECSLCs tumor growth *in vivo*. Compared with the SNX-2112 group and shSTAT3 group, the tumor weight and volume in the combination group were significantly reduced. The animal body in the combination group was not decreased, which suggested that the reduced tumor weight and volume were due to the effect of SNX-2112 with shSTAT3. Consistently, The Hsp90 client proteins *p*-ERK and *p*-AKT (Ser473) levels were reduced in the combination group. TUNEL assay demonstrated that the apoptotic effect was obvious in the combination group.

## conclusion

In summary, we demonstrated that STAT3 is overexpressed in clinical esophageal cancer samples by RNA sequencing. Combination treatment of SNX-2112 along with shSTAT3 inhibited ECSLCs proliferation, induced ECSLCs apoptosis and cell cycle arrest at G2/M phase, attenuated clonal growth, inhibited phosphorylation of Hsp90 client proteins, and decreased ECSLCs tumorigenicity. STAT3 overexpression reversed the anticancer effects of SNX-2112 in ECSLCs suggesting that the combination treatment of SNX-2112 with shSTAT3 suppresses ECSLCs proliferation through the STAT3 pathway.

## Data Availability Statement

The raw data supporting the conclusions of this article will be made available by the authors, without undue reservation, to any qualified researcher.

## Ethics Statement

The patients provided their written informed consent to participate in this study. The use of the clinical specimen for research purposes was approved by the Ethics Committee of Guangdong Food and Drug Vocational College.

## Author Contributions

D-dX designed the studies, carried out the experiments, performed statistical analyses, and wrote the manuscript; S-hC and Z-dZ read and revised the manuscript; P-jZ and YW analyzed the data and performed bioinformatics analyses. XW and H-qH conceived the study and analyzed the data; Q-yL and XX participated in the design and coordination of the study. Y-fW and RZ designed the study and participated in manuscript development. All authors contributed to manuscript revision and read and approved the submitted version.

## Funding

This study was supported by the National Natural Science Foundation of China Youth Fund (81702990), Guangdong Medical Research Fund (A2017312, A2018540, A2018238), Science and Technology Project of Guangzhou (201904010050). Natural Science Foundation of Guangdong Province (2017A030313449).

## Conflict of Interest

The authors declare that the research was conducted in the absence of any commercial or financial relationships that could be construed as a potential conflict of interest.

## Glossary

ABC: ATP-binding cassette
ALDH: aldehyde dehydrogenase
bFGF: basic fibroblast growth factor
CSCs: cancer stem cells
EAC: esophageal adenocarcinoma
ECSLCs: esophageal cancer stem-like cells
EGF: epidermal growth factor
ERK: extracellular signal–regulated kinase
ESCC: esophageal squamous cell carcinoma
ESCs: embryonic stem cells
HEEC: human esophageal epithelial cells
Hsp90: heat shock protein 90
IHC: immunohistochemical
JNK: c-Jun N-terminal kinase
PMSF: phenylmethylsulfonyl fluoride
shSTAT3: knockdown of STAT3
STAT3: signal transducer and activator of transcription 3
TICs: tumor-initiating cells
TUNEL: terminal deoxynucleotidyl transferase–mediated deoxyuridine triphosphate nick end labeling
17-AAG: 17-allylamino-17-demethoxygeldanamycin
