# Supplementary figures and images for: Suppression of Esophageal Cancer Stem-like Cells by SNX-2112 Is Enhanced by STAT3 Silencing
Source: Front Pharmacol. 2020 Dec 16;11:532395. doi: 10.3389/fphar.2020.532395 (PMC7772942; doi:10.3389/fphar.2020.532395)

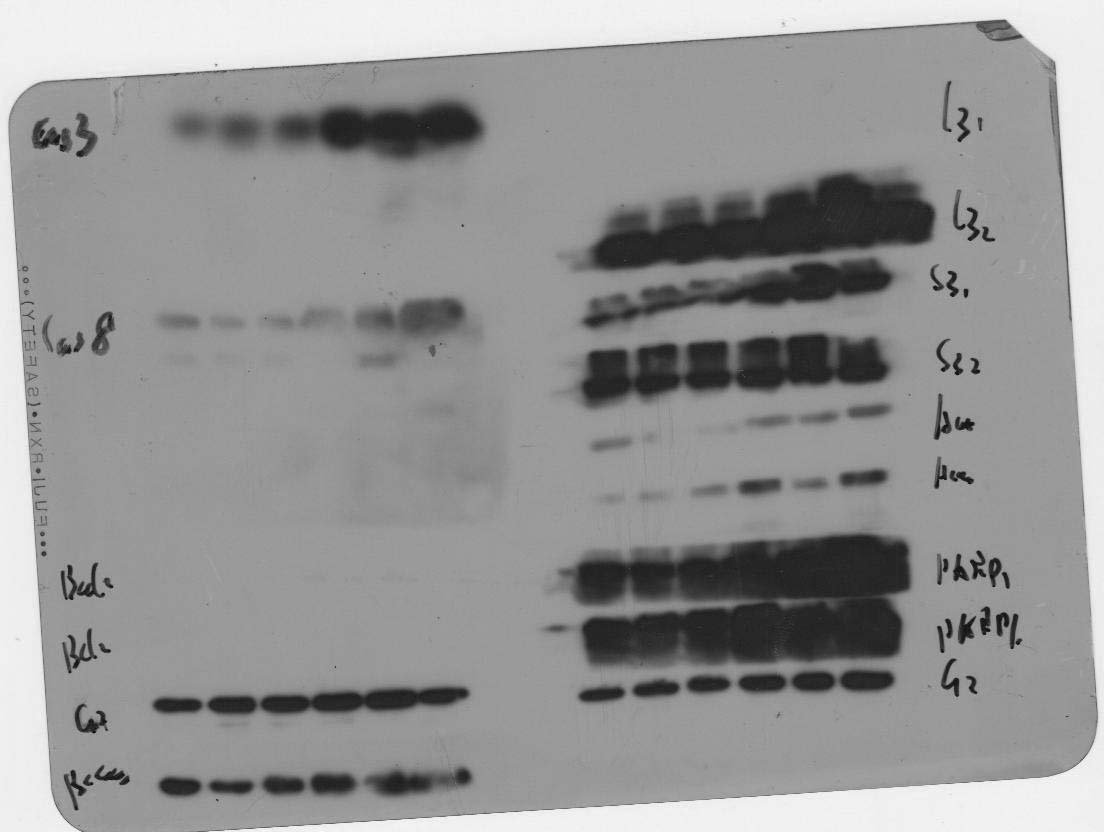

Supplement: Supplementary file 1 [file datasheet1.zip › original data of WB/Fig.1/1.jpg]

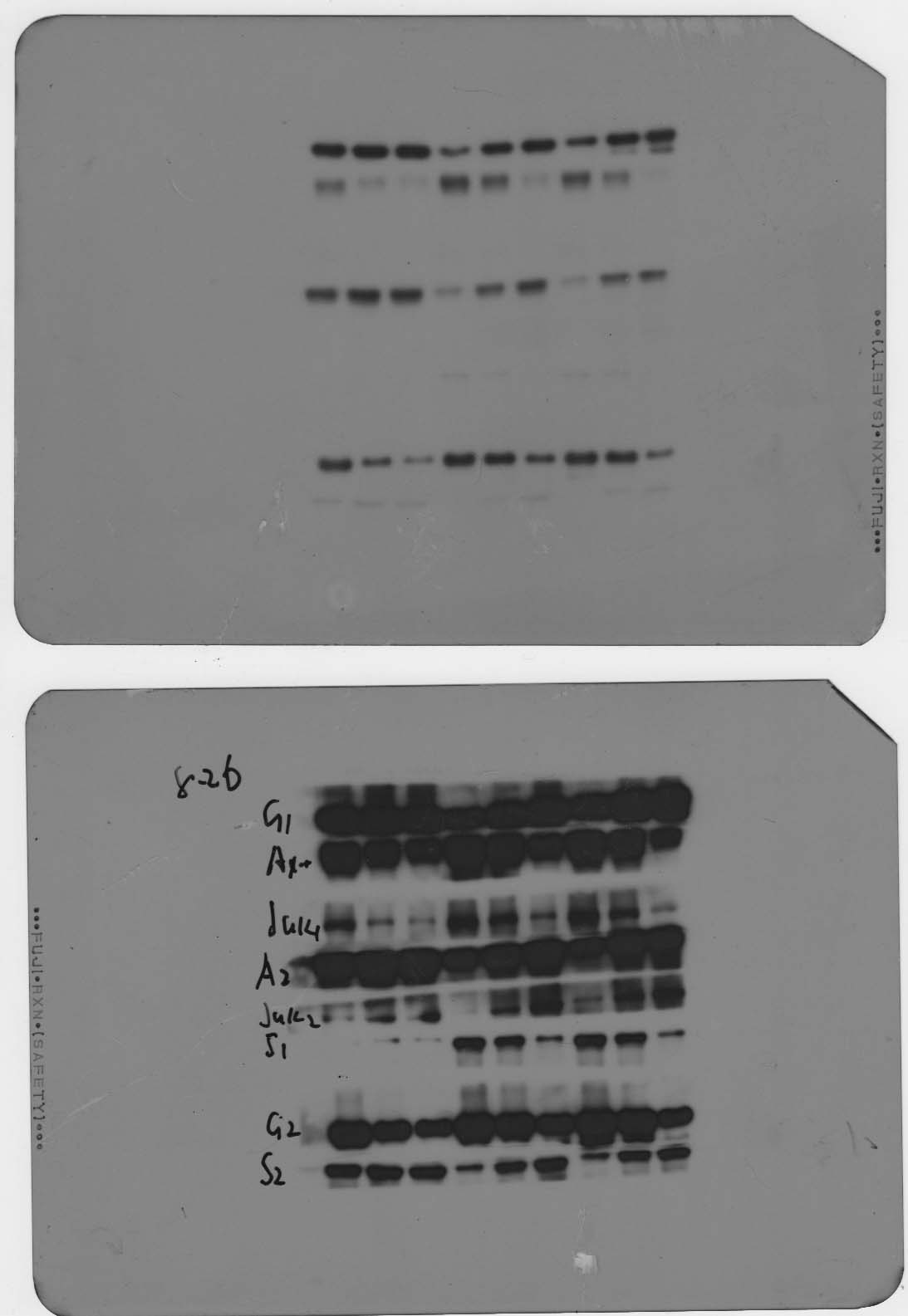

Supplement: Supplementary file 1 [file datasheet1.zip › original data of WB/Fig.5/1.jpg]

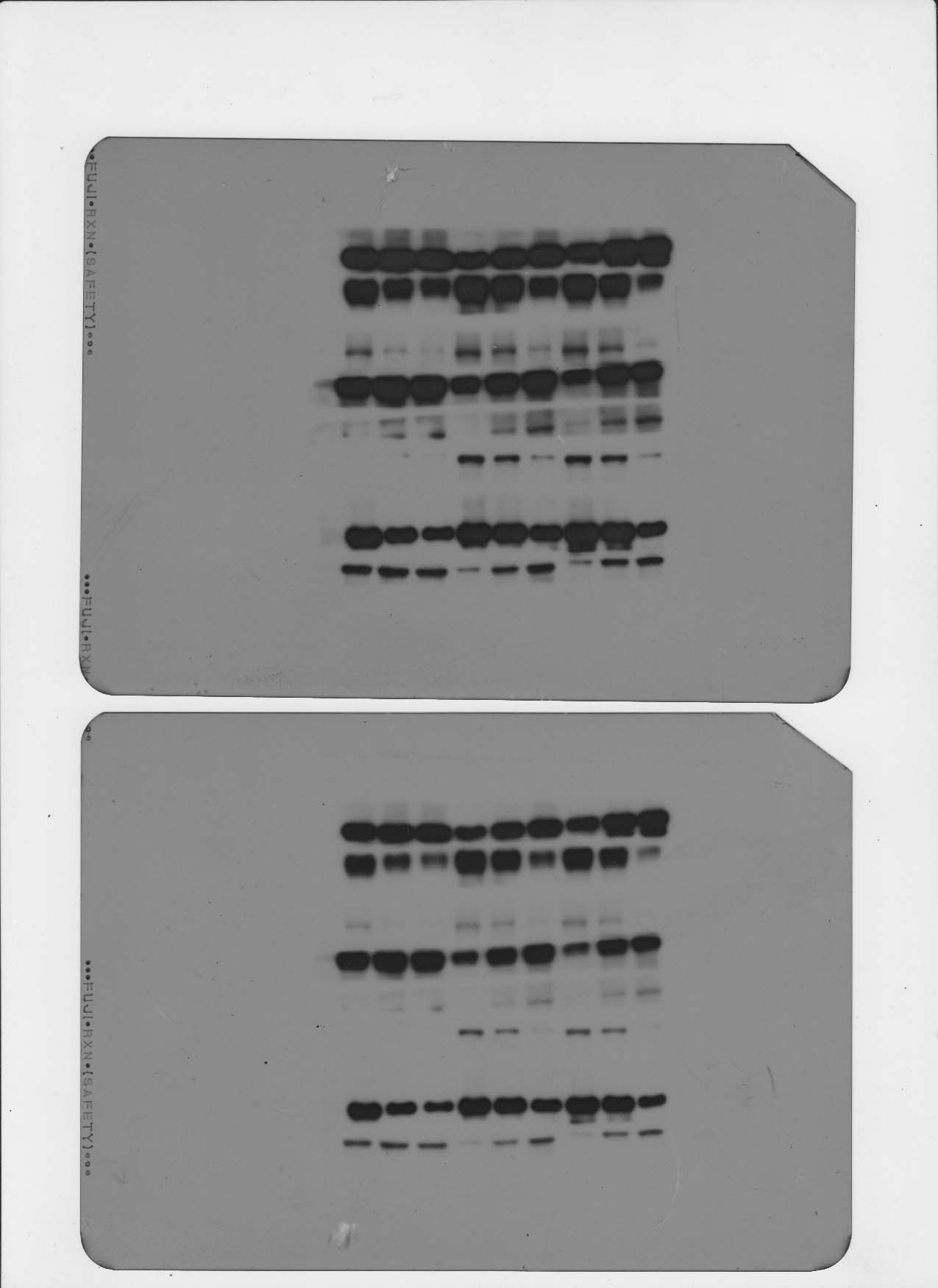

Supplement: Supplementary file 1 [file datasheet1.zip › original data of WB/Fig.5/2.jpg]
