# Supplementary material for: Suppression of Esophageal Cancer Stem-like Cells by SNX-2112 Is Enhanced by STAT3 Silencing
Source: Front Pharmacol. 2020 Dec 16;11:532395. doi: 10.3389/fphar.2020.532395 (PMC7772942; doi:10.3389/fphar.2020.532395)
Supplement: Supplementary file 2 [file datasheet2.doc]

SNX-2112 suppresses the proliferation of the esophageal cancer stem-like cells, which is enhanced by STAT3 silencing

Dan-dan Xu, Su-hong Chen, Peng-jun Zhou, Ying Wang, Zhen-dong Zhao, Xia Wang, Hui-qing Huang, Xue Xue, Qiu-ying Liu, Yi-fei Wang, Rong Zhang


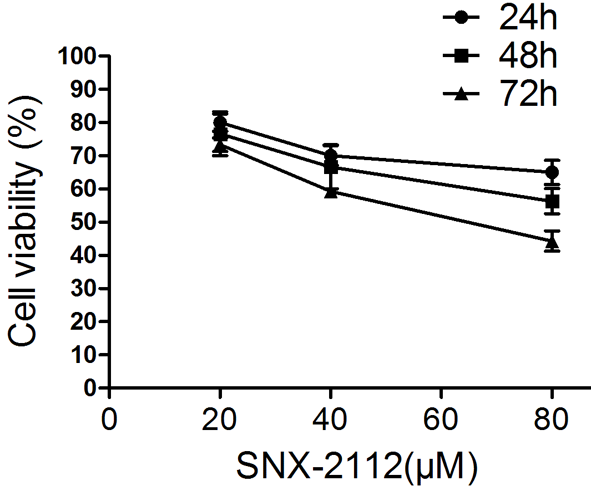


Fig.1


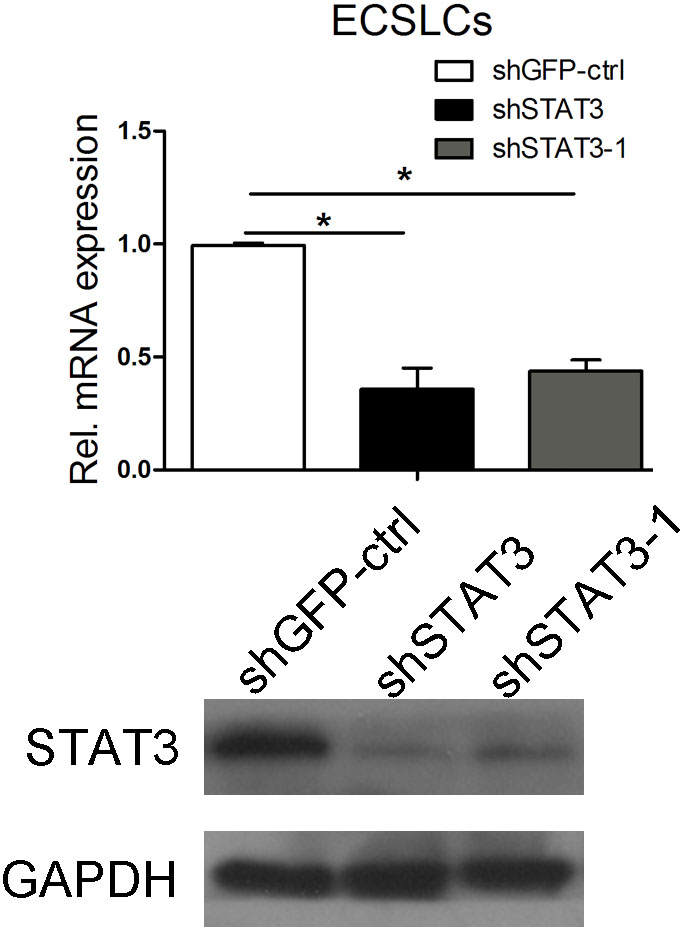


Fig.2

Summary of patients information

Numbers of age range (46-49) was 1, Numbers of age range (50-55) was 2, numbers of age range (56-59) was 1, numbers of age range (60-65) was 6, numbers of age range (66-69) was 1.

T classification Numbers of 2 cla. was 5 and numbers of 3 cla. was 6.

N classification Numbers of 0 cal. was 1, numbers of 1 cla. was 5, numbers of 2 cla. was 4, numbers of 3 cla. was 1.

M classification Numbers of M0 was 5, numbers of M1 was 6.

T tumor grade Numbers of G1 was 5, numbers of G2 was 4 and numbers of G3 was 2.

Location Numbers of middle was 4 and numbers of lower was 4 and numbers of upper was 3.

Abbreviations: T, tumor; N, node; M, metastasis.
